# Do One's Network Attributes Matter? Personal Networks of Older Adults and Self‐Rated Health: A Systematic Review

**DOI:** 10.1002/puh2.70455

**Published:** 2026-11-13

**Authors:** Kelemu Fenta Gebeyehu, Tong Yuying, Busha Taa, Jin Lei

**Affiliations:** ^1^ The Chinese University of Hong Kong Hong Kong China; ^2^ Department of Sociology University of Gondar Gondar Ethiopia

**Keywords:** convoy theory, older adults, personal network attributes, self‐rated health, sociocultural contexts, systematic review

## Abstract

Although there are mounting scholastic interests in examining personal network attributes and self‐rated health in later life, studies remain fragmented and inconclusive. This article systematically investigates current literature on the interpersonal worlds of older people and their association with self‐rated health. To address this, we searched the literature in four databases and grey literature using qualitative thematic analysis, adhering to PRISMA guidelines and registering with PROSPERO (CRD42022300858). We found that the social world of older individuals includes both structural and functional dimensions, such as contact frequency, network size, types of relationships, emotional closeness, and perceived social support. Researchers often focus on structural aspects, with typologies being a key concern. Older adults typically maintain diverse personal networks, which can be categorized into about 8 general and 20 specific forms, showing similarities across different demographics. The review also reveals that the personal networks of older individuals are associated with self‐rated health both positively and negatively. Although diverse networks generally support better health outcomes, restricted ties can have adverse effects. This review highlights the need to consider personal contexts to enhance the health and well‐being of older adults, contributing to ongoing debates and guiding policymakers in leveraging social networks for health.

## Graphical abstract

The article examines the current state of knowledge on the relationship between older adults’ personal environments and their self‐rated health. It identifies 8 general types and 20 specific categories of personal connections, emphasizing the varied health effects of these networks. Overall, diverse networks tend to correlate with better health outcomes, whereas more restricted networks are linked to poorer health outcomes. These findings highlight the significance of personal context in informing policy and intervention strategies.

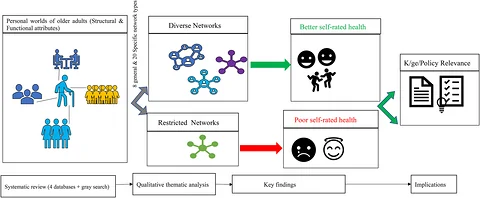

## 1 Background and State of Knowledge

The world population is aging rapidly, with nearly 2.1 billion people aged 60 and older [1, 2, 3]. This demographic shift signifies the importance of health and well‐being in later life, prompting research into the social determinants of self‐rated health (SRH) for this population [4]. Among these determinants, social relationships, mainly personal networks, are the key predictors of health and well‐being [5].

These networks, comprising diverse ties of family, friends, neighbors, and others, provide essential support to older adults, contributing to their well‐being [5, 6]. Decades of studies prove that socially integrated older adults report better SRH [7, 8]. Typically, for older adults facing diverse health challenges—physiological decline, retirement, and loss of their partners—personal networks are the primary sources of social integration, emotional, and instrumental support [1, 4]. The convoy theory argues that these effects are explained through the behavioral, psychological, and physiological mechanisms [6, 9].

However, many of the studies on social networks and SRH predominantly focused on structural network indices, including size, frequency of contact, or simply the number of living children [10, 11, 12]. However, recent research critiques this structural approach for overlooking the heterogeneous benefits and constraints that networks can have on health and well‐being [13, 14, 15, 16]. It also undervalues the comparable or independent predictive power of the qualitative aspects of personal networks for SRH [17]. Regardless of network size, limited high‐quality personal networks could have a significant influence on health by promoting autonomy and positive health appraisals [9]. Thus, scholars are providing additional evidence for the independent or integrative power of the non‐structural aspects of personal network attributes in shaping SRH in late adulthood [14, 18].

Although there are mounting scholastic interests in examining personal network attributes and SRH in later life, studies remain fragmented and inconclusive. Particularly, a systematic review is urgently needed to fill the following gaps: First, the absence of existing reviews that systematically address the comparative association between the multiple aspects of network characteristics and SRH within and across studies. Most scholars separately examine one or two network attributes, and it is difficult to comparatively address structural or/and/functional attributes that matter for SRH.

Although many studies confirm a connection between network types and older adults’ SRH, they often overlook which specific types of social ties contribute most to perceived health. A systematic review of these associations requires examining both the structures and functions of social ties and their health linkage [19].

Second, the current literature is very Western‐centric, focusing on limited individualist societies, and the link between personal networks and SRH of older adults is limited in collectivist cultures. Thus, given the potential uniqueness across contexts [20, 21], we are less informed about the differential or similar types of networks found across the two societies, and a cross‐cultural perspective is needed.

Finally, prior reviews focused on the link between network attributes and depression [22], cognitive functions [23], or mortality [24], but SRH, which is known as a powerful indicator of subjective health, was not the central focus of such studies. Given the distinctive dimension that SRH integrates the social, mental, and physical dimensions of health directly from the perspectives of the older adults, it warrants the current synthesis [25, 26, 27]. SRH, as the robust and holistic assessment of single or multiple aspects of one's overall health, has become a critical aspect of gerontological outcomes that predict disability, all‐cause mortality, health care utilization, later life capability, and resilience, regardless of the objective measure of health [25, 26, 27]. Hence, the understanding of the social determinants of SRH of the aging population is becoming a critical public health priority for local and global organizations.

Therefore, to address the gaps identified, the current study poses an essential question: Do the attributes of one's social network influence SRH among older adults? Specifically, guided by social convoy theory—which posits that humans are inherently social beings, connecting with others who provide various resources throughout their lives [9]—we aim to (1) describe the interpersonal landscapes of older adults in terms of network structures and functions that are associated with their SRH; (2) identify common types of social ties among older individuals within and across different countries; and (3) assess the relationship between the personal networks of older adults and their SRH.

## 2 Social Convoy Theory

It is noted that, as humans are socially wired with each other, their health is highly influenced by the diffusion of behaviors, ideas, attitudes, resources, tastes, and beliefs through such connections [4, 28]. To explain how personal networks influence the SRH of older adults, this review employs social convoy theory as a guiding framework. Thus, the theory is used as a guide to map the personal networks of older people across sociocultural contexts and identify the various network factors affecting their SRH. A convoy signifies the resources entrenched in social relationships highly linked to their members’ health and well‐being [29, 30].

As its central argument, the theory suggests that social ties with others are social resources through which individuals access material and immaterial resources important for health and well‐being throughout their life courses [9, 30, 31]. These health‐ and well‐being‐beneficial relational resources are assessed and explored through different mechanisms, and the hierarchical mapping technique is critical to identifying the resources or convoys available within the personal networks of older people [30].

This Technique identifies three layers of social convoys in the social networks of older adults over their lifetimes, based on their closeness and support potential [30, 32]. The core or closest layers represent the close ties that provide lifetime support, and such relationships are beyond material provisions and fulfillments of specific role requirements. Social network membership contexts in the second layer denote mildly intimate relationships within which the exchange of support transcends the fulfillments of roles. The final membership dynamics represent the older people's peripheral and temporal role‐based social ties as less stable than the previous two layers (see Figure 1).

**FIGURE 1:**
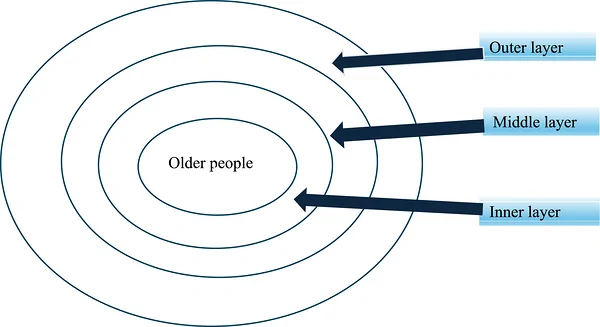
Hierarchical mapping of relational conveys of older people (adapted from [30]).

## 3 Methods and Materials

### 3.1 Search Strategy

This systematic review delves into the personal networks of older adults and their association with their SRH, encompassing perceived physical, psychological, and social well‐being. We thoroughly examined empirical studies using the latest Preferred Reporting Items for Systematic Reviews and Meta‐Analyses guidelines. The protocol for this systematic review has been registered on PROSPERO (CRD42022300858). We systematically retrieved papers from four databases: SCOPUS, PubMed, Age Line, and Sociological Abstracts (ProQuest). We considered all articles published up to December 2025 in our systematic search. Our search terms included descriptors related to networks, age, and health, such as (“personal network*” OR “social network*” OR “social ties” OR “social relationship*” OR “social contact”) AND (“health status” OR health) AND (“older adults” “elder* people” OR elderly OR older aged adults OR old age OR seniors OR “senior citizen*”). We narrowed our search to include only final peer‐reviewed articles and review papers written in English. We focused on documents about social networks or social support, older individuals or the elderly, aging, or health. We established clear screening parameters for including and excluding papers before and during the review process.

### 3.2 Inclusion and Exclusion Criteria

We reviewed articles based on their titles, abstracts, and key findings. We included studies that assessed the association between older adults’ networks and their SRH. Specifically, the inclusion criteria were as follows: (1) written in English, (2) focused on the personal networks of older adults, (3) included clear measures of the link between network attributes and SRH, and (4) the age of the older adults was 60 years and above. We excluded papers that mainly focused on social capital, social integration, or social support without addressing the social networks of older people and their SRH. Additionally, documents that focused solely on the relationship between personal networks and other outcomes were excluded.

### 3.3 Study Selection

After completing the search in the databases, we exported all the articles to an EndNote reference management system, and duplicates were removed. Then, we screened papers based on their title and abstracts. We managed the practical difficulties and differences in screening by reviewing the full articles. Inter‐reviewer disagreements were handled through constructive discussions and consensual agreements. Ultimately, the full texts of the final articles were downloaded for final inclusion and further processing.

### 3.4 Data Extraction and Thematic Analysis

We gathered data by distilling critical points from 15 articles that explored the association between various aspects of personal networks and the SRH of older adults. The information extracted included author names, publication year, study design and analysis methods, sample details, settings (community‐dwelling older adults or those in isolation centers), socio‐demographic characteristics of participants (age and gender), primary categories of personal networks (such as discussion, social support, and activity networks), measurement of personal networks (assessments of structural and functional characteristics), outcome variables, and key findings. These data were then utilized in the initial coding for thematic analysis, as presented in Figure 2.

**FIGURE 2:**
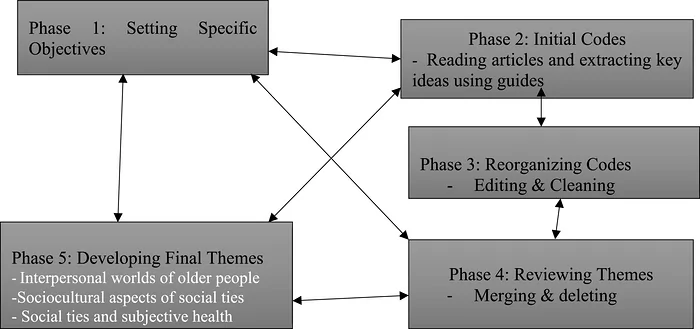
Thematic analysis framework.

We conducted the thematic analysis to analyze the extracted data manually using Microsoft Word. Notably, the main steps of thematic analysis effectively guided the identification and development of themes from the extracted documents. Using the extraction tools and review objectives as a framework, we generated coding themes through iterative reading of each article and data extraction protocols (refer to Figure 2). The codes were then refined and organized into broader themes through thorough editing and review within and across themes. Some codes were merged or removed to enhance diversity and comprehensiveness, developing higher level themes. Subsequently, we further categorized, refined, and named the themes. Ultimately, three themes related to the study objectives—the interpersonal worlds of older adults, the socio‐cultural context and social ties of older individuals, and the social network attributes and subjective health of older adults—were utilized to structure the review report accordingly.

### 3.5 Quality Appraisal

We evaluated the quality of each of the 15 articles using Kmet et al. quality assessment criteria for both quantitative and qualitative research [33]. Although the systematic search did not focus on specific analysis methods, all the included papers underwent quantitative analysis. The quantitative research assessment criteria consist of 14 items, 4 levels of assessment, and scores: no (0), partial (1), yes (2), and not applicable (3). The total quality rating for each study is determined based on the sum of possible scores; for more details, please refer to the source. According to the guidelines, the review had the following cutoff scores: ≤0.55 = low; >0.55 = medium; and ≥0.75 = high. As per these criteria, the reviewed papers were of medium or high quality, with almost all (13) being classified as high quality (refer to Table 1).

**TABLE 1:** Quality assessment criteria for quantitative studies (adopted from [33]).

1. Question/objective sufficiently described?
2. Study design evident and appropriate?
3. Method of subject/comparison group selection *or* source of information/input variables described and appropriate?
4. Subject (and comparison group, if applicable) characteristics sufficiently described?
5. If interventional and random allocation were possible, were they described?
6. If interventional and blinding of investigators was possible, was it reported?
7. If interventional and blinding of subjects was possible, was it reported?
8. Outcome and (if applicable) exposure measure(s) well defined and robust to measurement/misclassification bias? Means of assessment reported?
9. Is sample size appropriate?
10. Analytic methods described/justified and appropriate?
11. Some estimate of variance is reported for the main results?
12. Controlled for confounding?
13. Results reported in sufficient detail?
14. Conclusions supported by the results?

## 4 Results

The primary review processes are illustrated in the PRISMA diagram flow map (refer to Figure 3). The diagram outlines the article retrieval from four databases and random Google searches, removal of duplicate articles, papers remaining for full review, exclusion of articles for valid reasons, and the reports retained for analysis. A total of 1912 papers (1900 from databases and 12 from Google searches) were retrieved from four databases, namely, PubMed, SCOPUS, ProQuest (Sociological Abstracts), and Age Line, and sent to an EndNote library for further review. Eighty‐eight articles were removed as duplicates, leaving 1812 records for title‐ and abstract‐based reviews.

**FIGURE 3:**
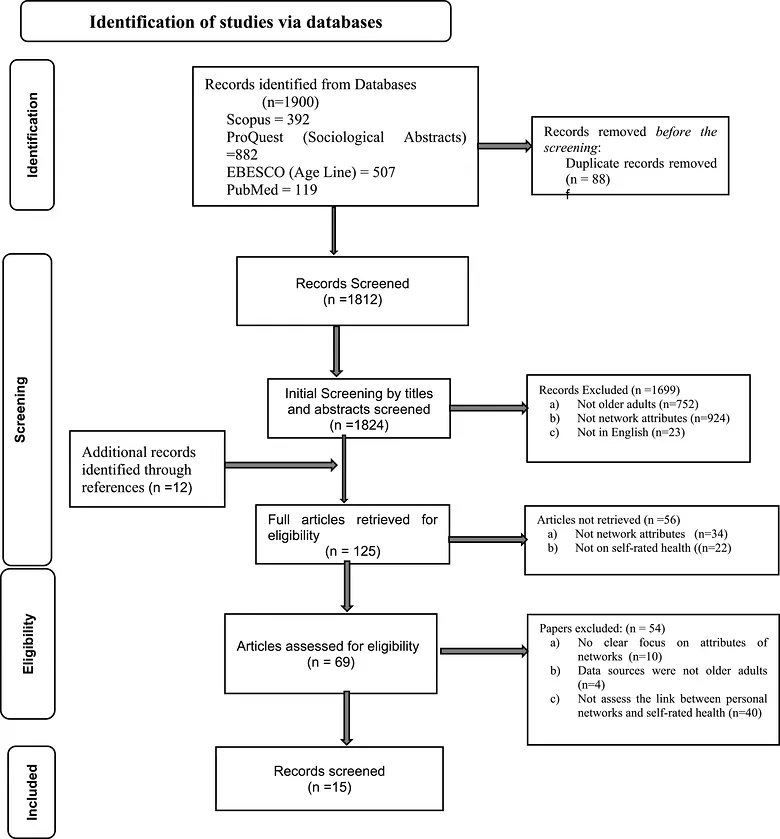
PRISMA 2020 flow diagram for systematic reviews [34].

Over half of the papers (1699) were excluded during the title and abstract screening stage, and 125 articles were retrieved for eligibility assessment. It is worth noting that papers were excluded on the basis of specific criteria: focus on SRH and nonnetwork attributes (*n* = 924) and non‐English language (*n* = 23). Around 752 papers were excluded during the initial phase, as their focus was on non‐older adults aged above 60, which is the commonly used age for older adults, though some countries might use an age below or above this [35]. Additionally, 12 relevant papers were obtained from Google Scholar, resulting in 125 full articles for final eligibility assessments. Fifteen studies were retained for systematic review, whereas 56 articles were excluded. Many of the excluded studies did not explore the association between personal network attributes and SRH of older adults (*n* = 40), focused on nonnetwork attributes (10), or utilized data sources not related to older adults (4). Finally, the findings of the final 15 papers were summarized and discussed (refer to Table 4).

### 4.1 Basic Features of Personal Networks of Older Adults

The analysis demonstrated that older adults have diverse forms of formal and informal networks. Notably, their social circles encompass various informal support networks [11, 29, 36, 37, 38, 39, 40, 41, 42], discussion [43, 44], and social participation and engagement in formal and informal activities [7, 45, 46].

As shown in Table 2, the 15 papers included sample sizes ranging from 195 [40] to 62,845 [44] among community‐dwelling older adults, with an average age of 74.5 years. These studies were conducted across four continents (Asia, North America, South America, and Europe), with more than half of the studies originating from Asia [7, 11, 38, 39, 40, 41, 42, 45], followed by Europe [43, 46], and North America [29, 37, 47].

**TABLE 2:** Major characteristics of the reviewed papers.

References	Methods	Sample	Settings	Sample characteristics		
				Age	Gender	Network category
[36]	Cross‐sectional regression analysis	3649	Community‐dwelling older adults	Above 60 (mean: 65)	M and F	Support networks
[49]	Longitudinal multinomial logistic regression		Older adults	Above 60 (mean: 67)		Discussion networks
[45]	cross‐sectional cluster, regression	4608	Older adults living with the community	Above 65 (mean: 72)	M and F	Discussion and social activity
[29]	Longitudinal cluster and multiple regression	1669	Noninstitutionalized seniors	Above 60 (mean: 70.4)	M and F	Support networks
[46]	Cross‐sectional regression	1890	Noninstitutionalized seniors	Above 65 (mean: 74.3)	M and F	Discussion and social participation
[7]	Longitudinal cluster	3501	Older adults living with the community	Above 65 (mean: 72.8)	M and F	Discussion and social participation
[11]	Cross‐sectional multiple classification	4573	Community‐dwelling older adults	Above 60 (mean: 70)	M and F	Support networks
[37]	Cross‐sectional cluster and regression	1462	Community‐dwelling older adults	Above 65 (mean: 69)	M and F	Support networks
[38]	Latent class analysis	6900	Community‐dwelling older adults	Above 65 (mean: 73.5)	M and F	Generic networks
[43]	Longitudinal study regression	14,101	Older adults living with the community	Above 65 (mean: 74)	M and F	Discussion networks
[39]	Cross‐sectional confirmatory factor	630	Older adults living with the community	Above 60 (mean: 65)	M and F	Support networks
[40]	Cross‐sectional cluster regression	195	Community‐dwelling older adults	Above 60 (mean: 70.4)	M and F	Support networks
[41]	Cross‐sectional latent class regression	9749	Community‐dwelling older adults	Above 65 (mean: 86)	M and F	Support networks
[44]	Cross‐sectional regression	62,845	Community‐dwelling older adults	Above 65 (mean: 71)	M and F	Contact networks
[42]	Cross‐sectional cluster regression	405	Community‐dwelling older adults	Above 60 (mean: 67)	M and F	Support networks

Regarding study design and methodological approaches, several scholars utilized cross‐sectional methods and employed various regression models to investigate the link between social ties and the SRH of older adults. Four articles [7, 29, 43, 47] used longitudinal analysis and were examined using multiple clustering and latent class analysis. Additionally, some papers incorporated variable‐centered and person‐oriented cluster analysis approaches [7, 29, 37, 38, 41, 45, 47].

Finally, the main body of the review is presented in line with the developed themes and specific objectives: personal network attributes, cross‐cultural contexts of personal network types embedding older adults in different cultural contexts, personal networks, and the SRH of older people.

### 4.2 Personal Network Attributes of Older Adults

The review reveals that the interpersonal worlds of older people comprise various network structures and functions that are relevant sources of material and nonmaterial support [50]. In‐line with the convoy theory, the lives of older people are surrounded by multiple formal and informal networks [30]. Scholars assessed the social networks of older people by asking them one or more of the following main questions: Enumerate 5–7 ties with whom you discuss important matters? [43, 47] How many times have you contacted tie members, including friends and neighbors? [36, 43, 44, 46] How many tie members do you have? [39], the types of personal ties [7, 11, 29, 37, 38, 39, 40, 41, 42, 45, 47], and what are/are the qualities of support received from your tie members? [43].

The assessment of important matter networks is one of the commonly used measures of personal networks of older adults with whom they discuss relevant matters over different periods, including months and a year [49]. Most of the studies incorporated the frequency of contacts as one key indicator of the social networks of older adults, used to uncover the number of contacts they have with their ties—neighbors, friends, family, and so forth (see Table 3). Though there are differences, they connect with their network members weekly and yearly. For instance, Jerliu et al. identified that older adults communicate with their social ties weekly and monthly [36, 46]. It is also recognized that older adults connect with their confidants yearly using physical and virtual mechanisms like phone calls and other virtual means [51].

**TABLE 3:** Social network types and attributes of older adults.

		Attributes and types of social networks	
References	Country	Structure	Function
[36]	Brazil	Contact frequency	Perceived supports
[49]	The United States	Network types	
[45]	South Korea	Network types: diverse married, diverse unmarried, diverse, activity‐focused, family‐focused, the restricted couple focused, restricted unmarried	
[29]	The United States	Network types: restricted (non‐family), non‐friends, diverse, family, friends	Perceived supports Perceived negative relations
[46]	Kosovo	Frequency of contacts, size	
[7]	South Korea	Types: Restricted, modern, friends, and diverse networks	
[11]	Israel	Types: Diversified, friends and neighbors, narrow family‐focused, religious, and attuned/restricted	
[37]	The United States	Diverse, friend, congregant, family, restricted	
[38]	South Korea	Types: Diverse family, diverse friend, friend‐focused, distant, and restricted	
[43]	Europe	Frequency of contacts	Emotional connectedness
[39]	India	Types: friends/neighbors, relatives/families, and confidant	Actual perceived support
[40]	Lebanon	Geographically distant youth, geographically close/emotionally distant, close family	Positive support and trust
[41]	China	Types: Private, coupled‐focused, non‐coupled‐focused, restricted	Supports support appraisal
[44]	South Korea	Frequency of contacts: neighbors, relatives, friends	
[42]	China	Types: Diverse, family, family restricted, friends, and restricted	Social supports

Regarding the modalities of their network ties, older adults connect with their social circles, mainly through face‐to‐face and, occasionally, online, and electronic outlets. The number of network members denotes the total number of ties older people have with multiple individuals, including confidants, families, and neighbors [29, 39]. The types of personal ties of older adults, which were assessed using diverse approaches, including latent class analysis, are commonly used personal network attributes in the reviewed studies [49].

Moreover, scholars also measured the personal networks of older adults using the different aspects of network functions, which denote the purposes, roles, and benefits that personal ties provide to individuals and groups [52]. In this vein, four aspects of social network functions are typical among older adults. The first category of network functions is related to the various forms of actual and perceived social supports (informational, emotional, affective, instrumental) exchanged and gained from network members [29, 36, 39, 40, 41, 42]. The other functional aspects of older adults are relationship quality (perceived hostile relations) that connotes the demanding nature of social ties [29], emotional connectedness [43], and trust between network members [40]. The functional aspects of ties signify the operations of networks to provide material and immaterial resources, and other opportunities for comparison and influence.

Therefore, it is widely recognized that elderly individuals' social connections encompass the overall count of their informal and formal network associates, diverse modes of in‐person and online interactions, emotional bonds with members, familial and nonfamilial network categories, tangible and intangible assistance within the networks, and favorable or unfavorable attitudes toward the social connections that encompass them.

### 4.3 Socio‐Cultural Context of Personal Network Types

The current review revealed that older individuals have diverse social network typologies exhibiting similarities and differences within and between countries and other personal backgrounds. Although there are variations in the classifications of personal networks, most studies have concentrated on specific elements of structural networks, particularly examining the link between network types and health and well‐being of older people [7, 11, 29, 37, 38, 39, 40, 41, 42, 45, 47].

Notably, we identified almost twenty network typologies common among older adults, which are mainly informal ties formed with neighbors, relatives, friends, family, and others (see Table 3). By critically assessing their nature of formation, we found eight common types of personal networks among older people: diverse or enriched (married, unmarried, family, friends, and mixed); friends (friend‐focused and non‐friend‐focused); family (religious family and narrow or family‐focused); couple (non‐couple‐focused and couple‐focused); private; activity‐focused; religious; and restricted (family, friends, married/couple‐focused, unmarried, and non‐family).

The diverse network typologies were the focus of almost all of the studies, except in the works of [39, 40]. These types of personal networks are characterized as having the most resourceful social ties, diverse members, frequent contacts, having better socioeconomic status than the remaining typologies, and actively engaging in social activities [38, 48]. These networks are prevalent among married and unmarried individuals, friends, and various other combinations, and they are gender‐specific. In contrast to the typically gendered aspects of socioeconomic circumstances, diverse social networks are more prevalent among older women than men. This is evident in how social ties are established among Korean women and older male adults Choi and Jeon [45].

Moreover, the religious networks of older adults mainly form ties with their family members for spiritual and other support but have weak ties with friends and others. Additionally, older adults with private networks are typically known to have minimal contact and support with and from siblings and children but have exceptional ties and support from community resources [41]. Older people with private networks are identified as having the lowest number of members and generate limited social support and health benefits. Older adults with different aspects of restricted networks are recognized as structurally engaged but socially disengaged, with the least number of social ties and engaged in limited social activities [38].

Finally, the personal network typologies of older adults show slight differences and similarities within and across countries, gender, and ethno‐racial identity. We found that the beneficial elements of personal networks are conditioned on their sociocultural contexts, where a typology beneficial in one place may not necessarily be crucial for health and well‐being [51, 52]. For instance, some empirical papers have shown that seemingly negative aspects of social ties, such as conflict‐arousing personal networks, are positively associated with health and well‐being [51, 53].

Though the diverse forms of friendship, family, and restricted social ties are common worldwide [29], there are differences within and across countries and continents. We identified that diverse and friend‐focused relationships are common in Western countries and that family‐focused ties are typical in Asian countries [7]. Diverse friendship networks in the United States are the dominant social ties [37], and restricted family‐personal social ties are common in some rural parts of China [42]. In rural India, older adults have non‐kin friendship networks rather than kin social ties with children and relatives [39]. In the case of South Korean older adults, friendship and restricted networks are the most stable types of personal relations [7, 38].

Moreover, the personal network typologies also differ by ethno‐racial identity and gender in the United States. In uncovering three types of personal networks of older adults—restricted, focused, and enriched—they Piedra and Iveniuk [49] found that these networks are evolving and their membership varies by ethno‐racial identity and gender. As relatively less mobile network types, the restricted and focused networks are common among Blacks and Hispanics. The enriched networks are becoming prevalent over time and are uncommon among females, Hispanics, and Blacks [49].

### 4.4 Personal Networks and SRH of Older People

The personal networks of older adults are identified as crucial predictors for SRH. (In this article, self‐rated health^1^ reflects the perceived judgment of older adults towards their physical, psychological, social, and functional aspects of health, not indicating morbidity and mortality outcomes.) In elaborating this argument, the social convoy perspective states that human beings are socially connected with their tie members, with whom they share ideas, behaviors, attitudes, tastes, and beliefs directly or indirectly related to their health conditions [9, 28].

In this vein, the review revealed that the various aspects of personal network attributes are significantly associated with the SRH of older people (see Table 4). For example, the frequency of contact, size, type, emotional closeness, and network support are closely linked to the self‐assessed health of elderly individuals. Caetano and his team clarified that low perceived social support and a small number of personal networks are connected to the poorer self‐assessed health of older adults in Brazil [36].

**TABLE 4:** Summary of findings and quality appraisal of the reviewed papers.

References	Outcome variable	Key findings	Quality appraisal score
[36]	Self‐rated health	Low perceived social support and limited connectedness are associated with the poor health of older women and men, respectively	0.86
[49]	Lonloiness	The different types of networks differently associated with self‐rated health where enriched and focused social networks with diverse others are associated with low loneliness than restricted networks	0.95
[45]	Mental health/self‐rated depression	Although men and women were affiliated with different social networks, older adults with diverse networks had better mental health than those with restricted ties	0.82
[29]	Self‐rated depression	Older adults with diverse networks had lower depression than those with non‐friend ties	0.86
[46]	Self‐rated health	Older adults who have frequent contact with their ties reported better self‐health than those with less frequent connections and no social engagements	0.77
[7]	Self‐rated well‐being	Those with limited ties have worse subjective health, depression, and life satisfaction than those with nonrestricted networks	0.95
[11]	Self‐rated health,	Resourceful networks, i.e., diversified friends and neighborhood networks, are strongly associated with better health of older people	0.77
[37]	Subjective health: loneliness, anxiety, and happiness	Older Americans have different types of social networks, and those embedded with diverse friendship and religious social ties have better subjective well‐being than seniors with restricted networks	0.73
[38]	Self‐rated health and depression	Older Koreans embedded with diverse friendship ties have better subjective health and lower depression than seniors with distant and restricted networks	0.82
[42]	Mental health	Lost and added confidants with close connections are strongly associated with the mental health of older adults	0.77
[39]	Self‐rated mental health	For Indian rural older adults, non‐kin friendship networks strongly predict their mental health/depression more than kin‐oriented social ties, including their children and relatives	0.77
[40]	Self‐rated mental health	Older adults linked with geographically and emotionally close families have better health functioning than those with socially and geographically distant family and non‐kin members	0.77
[41]	Subjective well‐being	Older Chinese adults reported two forms of family networks (coupled and non‐coupled) of which the coupled ties are the second common networks next to diverse ties. Older adults with diverse social ties had better physical health and well‐being than those with private networks	0.91
[44]	Self‐reported mental health	South Korean older people have contacts with their relatives, neighbors, and friends. Of all these ties, those who have frequent contact with their neighbors have the lowest depressive symptoms	0.73
[42]	Self‐rated health and depression	The study revealed a unique family‐restricted network among Chinese rural older adults. Older adults with diverse and friend networks had lower depression and better self‐rated health than those with restricted networks	0.86

Similarly, older adults with non‐friends and diverse networks reported higher and lower levels of perceived depressive symptoms, respectively [29]. These conclusions are partly due to the valuable nature of such networks in terms of the quality of perceived support and trust [40]. Individuals with supportive and resourceful social networks are considered important sources of health‐promoting material and nonmaterial resources [56].

Moreover, the different types of personal networks are associated with various health outcomes of older adults. For instance, the review revealed that older adults with diverse networks reported better subjective health than those with restricted social ties [29]. Older Americans with diverse and religious ties reported better subjective well‐being and lower depression than older people with restricted networks [29, 37].

Interestingly, the association between the various types of personal networks and SRH of older adults differs by ethno‐racial identity, gender, and context. In the case of South Korea, even though restricted types of social ties are related to poor health for men and women, family‐focused and social relations are associated with more significant depressive symptoms for older women than men [45]. The links between personal network types and SRH of older adults are gendered in Brazil, where older women with family‐focused social ties and men and women older adults with restricted, unmarried, and couple‐focused social ties reported higher depressive symptoms [45]. In the same vein, as a relatively less mobile network type, the restricted and more mobile focus networks are common among females, Blacks, and Hispanics and linked with poor SRH [49].

Additionally, South Korean older people with limited and distant social ties reported worse SRH than those embedded with nonrestricted family and diverse friendship networks [7, 38]. In the context of Israeli older adults, those with diverse neighborhood and friendship social ties reported better SRH than those with religious, narrow, family‐focused, and restricted networks [11]. It has been identified that in China, older adults with diverse social ties reported better SRH than those with private and restricted networks [41, 42]. In other parts of Asia, non‐kin friendship networks are associated with higher depression than kin social ties with their children and relatives [39]. On the contrary, in the context of Lebanon, close family ties are significantly linked with the functional and mental health of older adults [40].

Apart from the types of networks, other attributes like the frequency of contact and network size have a known association with the SRH of elderly people. Notably, Jerliu et al. noted that although Kosovan older adults have limited social engagements in group activities, their frequent contact with families and friends is more positively associated with health than those with few social networks and those with no social engagements [46]. On the same note, South Korean older people who make frequent contact with their relatives, neighbors, and friends reported lower depressive symptoms; above all, those who have weekly contact with their neighbors reported lower depressive symptoms than those who rarely contact their neighbors, friends, and relatives [44].

Though understanding the association between the structural and/or functional aspects of networks of older individuals and their self‐reported outcomes is a critical area of research, the more salient aspects are still very limited. Schwartz and Litwin suggest that the quality of relationships is more significant than their structure. Specifically, the addition or loss of confidants with solid connections is linked to improved or diminished mental health in older adults, respectively [43].

## 5 Conclusion and Discussion

We uncovered that the interpersonal world of older people comprises structural and functional dimensions, including frequency of contact, size, and types of social relationships, emotional closeness, and actual and perceived social support. The scholars predominantly focus on the structural aspects of personal networks of older adults, of which the typologies were the main concern of several studies.

Most importantly, older adults maintain many types of personal networks comprising approximately eight general and twenty specific forms, which have similarities within and across countries, gender, and ethno‐racial identity. For instance, it is common for older South Korean adults to have restricted social networks [7, 38, 55]. However, it is important to note that these restricted ties are not universal among all older Korean adults; they are limited to those with restricted ties formed with friends, relatives, and family [7, 38, 55].

Similarly, Fiori et al. [50] identified that diverse‐supported, family‐focused, friend‐focused–supported, friend‐focused–unsupported, restricted–non‐friends–unsatisfied, and restricted–nonfamily–unsupported networks are typical social connections among older adults in Berlin, Germany. Moore et al. also identified that, in the context of Africa‐Togo, older adults living with HIV/AIDS are mainly socially connected with their relatives and non‐kin individuals [58]. Other scholars also identified that diverse friends, family, and restricted ties are significant personal network attributes of older adults [29, 38, 45, 48].

It is also clear that personal networks of older people can have protective and detrimental associations with their SRH outcomes. Typically, the association between social connections and SRH of older adults varies depending on the types of personal networks. Diverse social connections are positively associated with SRH, providing access to beneficial resources [59]. Conversely, restricted social networks are linked to adverse health outcomes, as they can lead to isolation and a lack of support, ultimately resulting in poorer SRH [40, 41, 58].

Finally, our review also confirmed the existing research gaps and scholarly debate on whether the structural or functional aspects of personal network attributes are linked with the self‐reported health of older people. For instance, some researchers posit that functional elements, such as the composition of personal networks, are significantly associated with SRH more than structural elements [43]. They have noted that the absence of confidants has the same effects on mental health as gaining or losing confidants with weak connections [43].

## 6 Limitations and Future Directions

Although the understanding of the typical personal network structures and functions and the sociocultural context of these attributes and their linkage with SRH among older adults provides valuable inputs for future studies, our review has the following gaps that warrant further investigation.

First, though we described the structural and functional aspects of personal network attributes and their association with the SRH of older people, a detailed review is needed to uncover whether the structural or/and/or functional aspects of personal worlds of the older population matter for health and well‐being.

Second, as the current review investigates the sociocultural contexts of older people's personal network typologies and their association with SRH, future reviews can uncover comparisons with different dimensions and the mechanisms behind the similarity and/or dissimilarity across cultures, sociodemographic backgrounds, and time.

Finally, although the current study describes the association between diverse aspects of personal network attributes and SRH of older adults, we need other scholars to review the mechanisms and variations across place and time to analyze the causal relationships between social network dynamics and various health outcomes over time.

## Author Contributions

**Kelemu Fenta Gebeyehu**: conceptualization, investigation, writing – original draft, methodology, validation, writing – review and editing, software, formal analysis. **Tong Yuying**: methodology, writing – review and editing, supervision. **Busha Taa**: supervision, writing – review and editing. **Jin Lei**: writing – review and editing, methodology, supervision.

## Funding

The authors have nothing to report.

## Ethics Statement

This systematic review adhered to the latest guidelines of Preferred Reporting Items for Systematic Reviews and Meta‐Analyses. It is essential to highlight that ethical approval from PROSPERO was obtained, and the review was registered at CRD under reference number 42022300858.

## Conflicts of Interest

The authors declare no conflicts of interest.

## Data Availability

The data used to produce these results are readily available as part of the review paper, and additional data are unnecessary.

## Footnotes

1 In this paper, self‐rated health reflects the perceived judgment of older adults towards their physical, psychological, social, and functional aspects of health, not indicating morbidity and mortality outcomes
